# Development and Evaluation of a Web-Based Platform for Personalized Educational and Professional Assistance for Dementia Caregivers: Proposal for a Mixed Methods Study

**DOI:** 10.2196/64127

**Published:** 2024-08-07

**Authors:** Logan DuBose, Qiping Fan, Louis Fisher, Minh-Nguyet Hoang, Diana Salha, Shinduk Lee, Marcia G Ory, Tokunbo Falohun

**Affiliations:** 1 School of Public Health Texas A&M University College Station, TX United States; 2 Olera lnc Houston, TX United States; 3 Department of Public Health Sciences Clemson University Clemson, SC United States; 4 School of Medicine Texas A&M University College Station, TX United States; 5 College of Nursing University of Utah Salt Lake City, UT United States; 6 Department of Biomedical Engineering Texas A&M University College Station, TX United States

**Keywords:** Alzheimer disease, dementia caregivers, digital platform, financial planning, legal planning, usability study, evaluation, older adults, caregiver support

## Abstract

**Background:**

Alzheimer disease (AD) and AD-related dementia are prevalent concerns for aging populations. With a growing older adult population living in the United States, the number of people living with dementia is expected to grow, posing significant challenges for informal caregivers. The mental and physical burdens associated with caregiving highlight the importance of developing novel and effective resources to support caregivers. However, technology solutions designed to address their needs often face low adoption rates due to usability issues and a lack of contextual relevance. This study focuses on developing a web-based platform providing financial and legal planning information and education for dementia caregivers and evaluating the platform’s usability and adoptability.

**Objective:**

The goal of this project is to create a web-based platform that connects caregivers with personalized and easily accessible resources. This project involves industrial, academic, and community partners and focuses on two primary aims: (1) developing a digital platform using a Dementia Care Personalization Algorithm and assessing feasibility in a pilot group of caregivers, and (2) evaluating the acceptability and usability of the digital platform across different racial or ethnic populations. This work will aid in the development of technology-based interventions to reduce caregiver burden.

**Methods:**

The phase I study follows an iterative Design Thinking approach, involving at least 25 dementia caregivers as a user feedback panel to assess the platform’s functionality, aesthetics, information, and overall quality using the adapted Mobile Application Rating Scale. Phase II is a usability study with 300 dementia caregivers in Texas (100 African American, 100 Hispanic or Latinx, and 100 non-Hispanic White). Participants will use the digital platform for about 4 weeks and evaluate its usefulness and ease of use through the Technology Acceptance Survey.

**Results:**

The study received funding from the National Institute on Aging on September 3, 2021. Ethical approval for phase I was obtained from the Texas A&M University Institutional Review Board on December 8, 2021, with data collection starting on January 1, 2022, and concluding on May 31, 2022. Phase I results were published on September 5, 2023, and April 17, 2024, respectively. On June 21, 2023, ethical approval for human subjects for phase II was granted, and participant recruitment began on July 1, 2023.

**Conclusions:**

Upon completing these aims, we expect to deliver a widely accessible digital platform tailored to assist dementia caregivers with financial and legal challenges by connecting them to personalized, contextually relevant information and resources in Texas. If successful, we plan to work with caregiving organizations to scale and sustain the platform, addressing the needs of the growing population living with dementia.

**International Registered Report Identifier (IRRID):**

DERR1-10.2196/64127

## Introduction

### Background

Currently, 6.7 million Americans live with Alzheimer disease (AD) and AD-related dementia, a progressive and debilitating neurocognitive disease that leads to loss of memory, motor function, and other psychological symptoms [1]. With the growing aging population in the United States, the number of people living with dementia is projected to grow exponentially to 13.8 million by 2060 [1]. These trends indicate a growing public health crisis that requires multifaceted interventions. People living with dementia are primarily cared for by informal caregivers, typically spouses and adult children [2,3], with as many as 16 million Americans currently serving in this role [4]. These informal caregivers of people living with dementia often have a higher risk of developing depression, anxiety, social isolation, and physical problems due to the chronic stress and diverse burdens associated with caregiving [5,6]. In recent years, dementia caregivers are estimated to provide more than 18 billion hours annually with an estimated economic value of US $346 billion for their care services [1], and this informal care cost is projected to increase to US $2.2 trillion in 2060 [7].

A large part of the informal caregiver burden is related to various responsibilities in caregiving for people living with dementia, including management of the care recipient’s financial, legal, and estate-related challenges [6,8]. Without adequate support, caregivers often struggle to find or use proper resources, making even simple tasks burdensome. Furthermore, these primary family caregivers often must balance the challenges of caregiving with other personal responsibilities, such as employment and family obligations [8,9]. Considering the significant financial burden associated with caregiving, role strain is further exacerbated when caregiving requires a full-time commitment. Six out of 10 family caregivers of people with dementia have reported significant work impacts ranging from reduction of work hours to early retirement due to their caregiving obligations, which disrupts their wages and employment and depletes their savings [6,10]. The varied progressive nature of dementia further compounds the complexity of care for people with dementia, increasing the psychological, physical, and financial factors of the caregiving burden [1,11].

The variation in caregiver burdens related to sociodemographic characteristics contributes to greater complications. Burdens are often exacerbated in ethnic and minority populations, who generally have higher rates of dementia but lower access to caregiving services and information [12,13]. In addition, ethnic minorities can experience complications when communicating with care professionals due to cultural or linguistic barriers [13]. These barriers can disproportionately affect nonnative English speakers, increasing the informal caregiver burden due to the greater experienced inaccessibility in support systems within the American health care system. Unique stressors have also been demonstrated in gender or sexual minority caregivers, further emphasizing the diversity of caregiving experiences and challenges within the United States [14]. Literature has shown that considering unique barriers or challenges in diverse demographics when developing support interventions increases their effectiveness in reducing caregiver burden [15].

### Technological Interventions and Artificial Intelligence–Driven Digital Health

There are many professional caregiver assistance organizations that provide services to informal caregivers, including aging care law, financial services, respite care, in-home care, older adults living, and medical services [16]. Digital interventions present a promising tool for assisting caregivers in identifying relevant professional caregiver assistance organizations services. A myriad of digital interventions have been developed to aid caregivers of people with dementia in identifying these services, ranging from web-based training, educational forums, caregiving support groups, and videoconferencing [17-19]. However, the adoption rate of these technologies remains low outside of pilot studies. This low adoption rate can be attributed to factors such as poor usability and accessibility, information complexity, funding limitations, and lack of contextualized relevance [8,20-24]. This issue is especially pronounced among minority populations, such as African American and Hispanic or Latinx caregivers, where cultural differences and caregiving challenges are often not considered in the development and evaluation of technology-based interventions [25].

Factors influencing the implementation and adoption of technology-based interventions by informal caregivers include the expected or perceived value by users, the features of the technology, the characteristics of the caregivers, and the condition of dementia [26]. According to the Technology Acceptance Model (TAM), perceived usefulness and ease of use are the 2 most important factors in determining how likely users are to adopt or reject new technologies [27]. The features of developed technologies, such as physical appearance, simplicity, and usability, are crucial for caregiver adoption [26]. In addition, the continued adaptation of technology over time, as dementia progresses and as caregiving needs evolve, is noted as important [26]. Family caregivers come from various racial and ethnic groups, generations, occupations, financial situations, and educational and cultural backgrounds [6], and their characteristics and specialized care needs are widely mentioned as influencing factors for technology adoption [26].

Artificial intelligence (AI) can offer various benefits to developing interventions that help caregivers better understand and use information in real time, leading to a more personalized and effective decision-making process [28]. AI has the potential to improve perceived usefulness and ease of use by personalizing and simplifying complex information. For instance, AI-driven applications can often offer user-friendly interfaces and navigation, reducing the learning curve for caregivers who may not be technologically savvy and enhancing user experience. Moreover, AI can be used to create and deliver personalized dementia care suggestions, meeting the evolving needs of people with dementia and their caregivers [29].

### Development of a Caregiving Support Platform

While there is increasing interest in technology interventions for dementia caregiving, limited literature has explored their effectiveness outside of research settings. Early involvement of caregivers, as emphasized in community participatory research and dissemination and implementation science, can enhance intervention adoptability and sustainability [18,30-32]. Our study seeks to address the paucity of research by exploring the efficacy of delivering caregiving resources and services in a technology-driven setting that considers individualized and cultural issues, in order to provide personalized, relevant, timely interventions to caregivers of people living with dementia.

To address this gap, our team developed a website-based platform designed to provide personalized caregiver support, including identification of caregiving resources related to older adults living arrangements, financial services, and legal services, as well as education related to dementia management and caregiving. In our web-based care planning tool, the Olera.care platform, we use our Dementia Care Personalization Algorithm (DCPA) based on logic decision trees and geolocation to provide caregivers of people living with dementia with a tailored guide on the aging care, older adults living, financial, and legal aspects of dementia caregiving (Olera.care). Studies have shown that such caregiving assistance is particularly effective in reducing caregiver burden [8]. To provide a technological intervention that would reduce the caregiving burden and address the needs of caregivers of people living with dementia, we developed a study with 2 phases centered in a community-engaged approach [33,34].

In further development cycles, various AI elements will be incorporated into our digital platform to take advantage of the rapidly advancing technology to increase acceptance and adoption. These AI elements include large language models (LLMs), a novel class of AI, and personalized care planning agents specialized in social assistance functions and resource connection. LLMs are a type of AI program that can recognize and interpret vast amounts of human language, texts, and data. Fine-tuning of LLMs through aging care domain-specific training can vastly improve algorithm accuracy and provide the most up-to-date information that is relevant for each user, enabling caregivers to navigate and use preexisting resources and information databases more effectively. The LLM’s capacity is particularly valuable in navigating the complexity of dementia caregiving information, especially for individuals unfamiliar with financial and legal terminology, those unfamiliar with terminology related to aging services, or those who are uncertain about their specific needs. In addition, personalized care planning agents, capable of perceiving information and making decisions, can be integrated to guide users through various resources and instructions for navigating websites, significantly improving the user interface and user experience. These agents can be optimized using machine learning, specifically through reinforcement learning from human feedback that leverages domain expertise to train the AI to output more contextually relevant responses.

### Evaluation Metrics and Framework

There are several metrics to evaluate the usability, ease of use, and acceptance of health care technological interventions, including the Mobile Application Rating Scale (MARS) and the TAM [35-37]. The MARS evaluates a digital product based on the functionality, design, information quality, engagement, and subjective quality of digital applications using a 5-point Likert scale survey [35]. The TAM evaluates the acceptability of a technology primarily based on its perceived usefulness and perceived ease of use among users [36,37]. Perceived usefulness refers to the degree to which a person believes that using a particular system can allow them to work more quickly, fulfill its intended purpose, increase productivity, enhance effectiveness, and make the job easier, with responses ranging from “strongly disagree” to “strongly agree” [38]. Similarly, perceived ease of use indicates the degree to which a person believes that the system is easy to learn, controllable, clear and understandable, flexible, easy to become skillful, and easy to use [38]. According to the TAM, perceived usefulness and ease of use are critical factors influencing the adoption or rejection of new technologies, and it has been used to determine the likelihood of dementia caregivers adopting digital technology interventions [39]; therefore, it is particularly suited for this evaluation study.

### Study Objectives

This study has 2 phases to develop, evaluate, and refine the Olera.care web-based platform. Phase I aims to develop the Olera.care platform, identify the caregiving challenges and needs of informal caregivers, and pilot-test the performance of the platform among a group of dementia caregivers. Phase II aims to understand the perceived usefulness and ease of use of the Olera.care platform among 3 different sociodemographic groups of informal caregivers of people living with dementia: African American, Hispanic or Latinx, and non-Hispanic White American.

## Methods

### Study Overview

The study consists of 2 phases of platform development and evaluation (Textbox 1). Phase I of the study involves identifying caregiving challenges and needs, developing the Olera.care web-based platform, and pilot-testing the usability of the platform in a group of dementia caregivers. Phase II aims to evaluate the Olera.care platform among 3 of the largest racial and ethnic groups in Texas and iteratively develop the Olera.care platform. Human subjects research was critiqued by the National Institutes of Health reviewers with key comments (Multimedia Appendix 1) addressed including clarifying risks and benefits for human participants in informed consents, along with privacy protection protocols.

**Study overview**

*Phase I: Platform development and pilot test*

Development of platform (Build Stage)

- Task 1: Compile care resources and educational materials
- Task 2: Build a logic decision tree for personalized recommendations
- Task 3: Design user questionnaire to collect user characteristics and develop prototypes
- Task 4: Develop a web-based application

Pilot testing of the platform in caregivers (n=30)

- Qualitative research: Identify caregiving challenges and needs for the platform using one-on-one interviews with caregivers
- Quantitative research: Collect user characteristics and preliminarily evaluate the platform using modified Mobile Application Rating Scale

*Phase II: Iterative platform development and evaluation*

Iterative development of the platform

- Expand care resources database outside of Texas
- Update educational materials of Alzheimer disease and Alzheimer disease–related dementia
- Integrate phase I feedback to platform features

Usability study across different racial or ethnic groups (n=300)

- Develop a comprehensive evaluation survey using the technology acceptance model
- Enroll and instruct caregivers to complete tasks:
  1. Generate a personalized caregiving checklist
  2. Identify and save 3 relevant resources to “favorite” list
  3. Register for “recommendation of the Day” and complete technology acceptance survey (TAS)
  4. Four-week interaction with the platform followed by a second completion of the TAS
- Evaluate perceived usefulness and ease of use of the platform across caregiver characteristics

### Platform Development

The development of the platform (Olera.care) adopts a build-measure-learn framework to prioritize the needs and preferences of caregivers for people living with dementia, using the Design Thinking product design methodology [40]. This iterative development process consists of three major steps: (1) prototypes will be developed to address preidentified user needs [Build Stage], (2) product usability will be tested [Measure Stage], and (3) key lessons will be identified for changes in next product iteration [Learn Stage]. The platform uses user feedback and a self-improving DCPA to provide caregivers with tailored educational information on the legal, financial, and estate planning aspects of dementia caregiving, as well as details on relevant local care and support options. DCPA considers care recipients’ stage of disease, financial circumstances, and care preferences to create unique recommendations. As a result, when the platform is in use, information is presented in a personalized and user-friendly manner that enhances the experience of caregivers when searching online for Alzheimer disease and AD-related dementia (AD/ADRD) support and reduces time spent sorting through general search engine results. We are improving the DCPA algorithm by leveraging LLMs and personalized AI agents. These AI agents will enhance our ability to provide real-time support, guidance, and information to caregivers to find dementia care services. By learning from caregivers’ interactions with Olera.care, these AI agents will continuously improve in delivering personalized and contextually relevant assistance.

**Figure 1:**
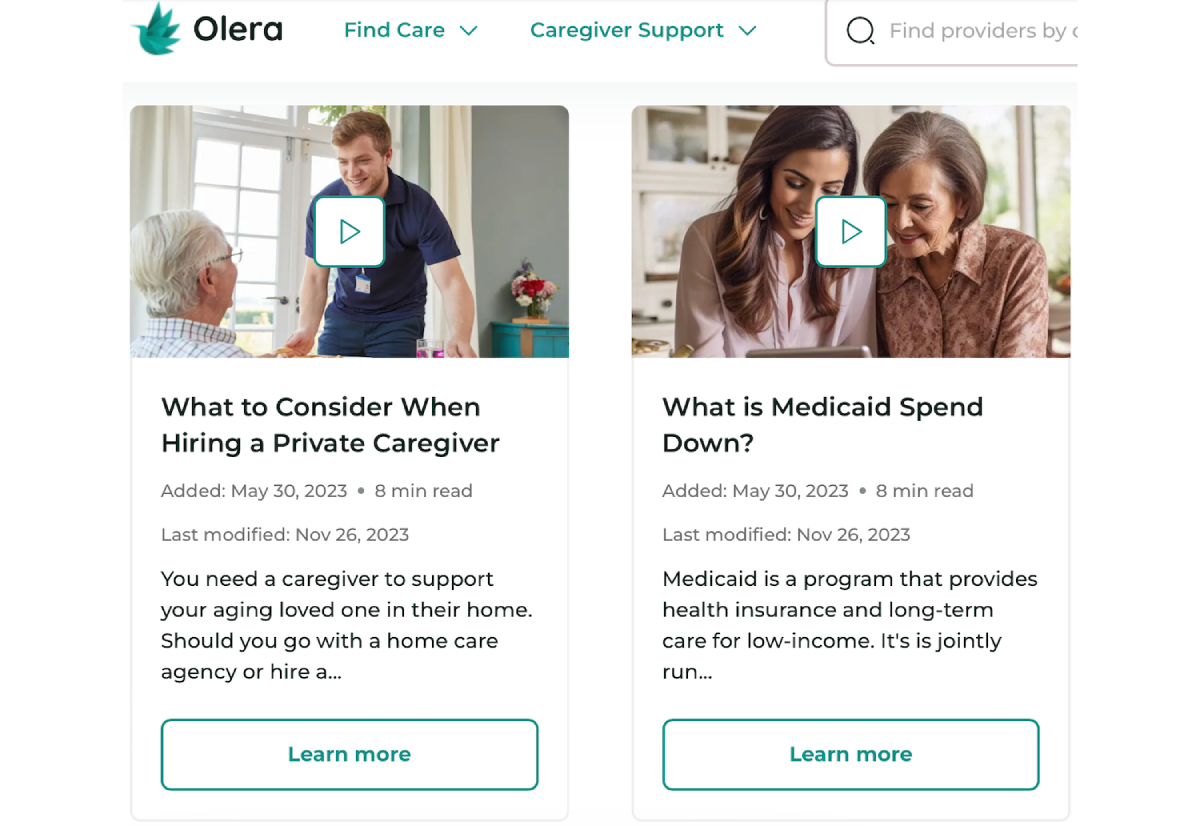
Video educational materials on the Olera.care platform.

**Figure 2:**
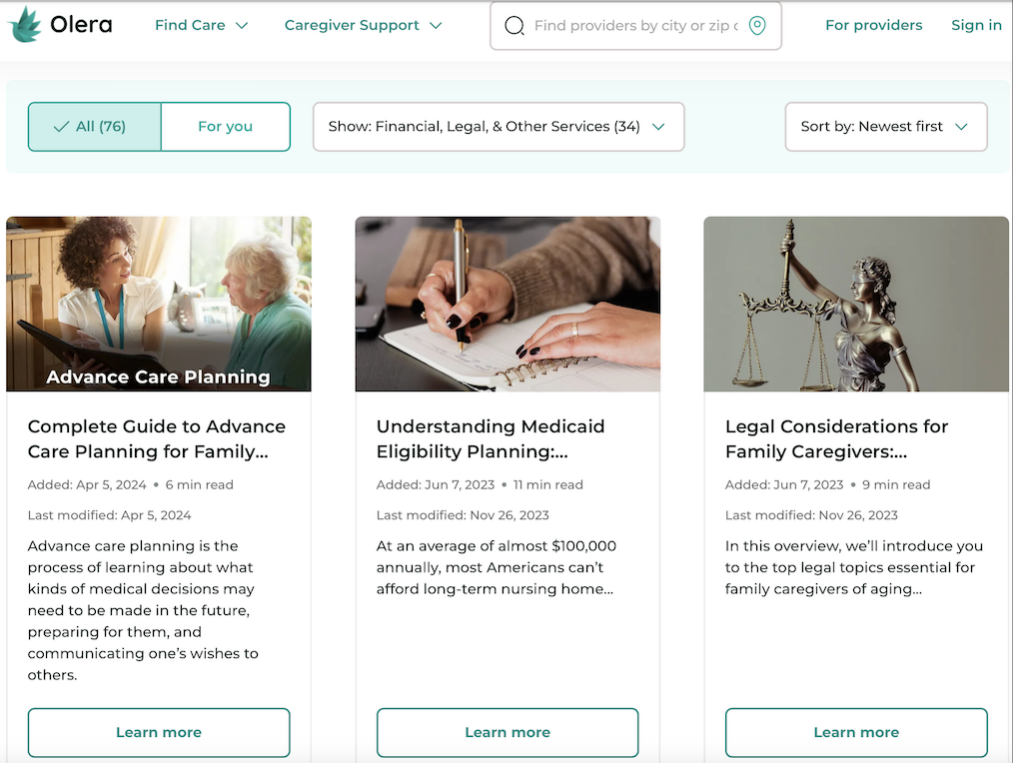
Article educational materials on the Olera.care platform.

**Figure 3:**
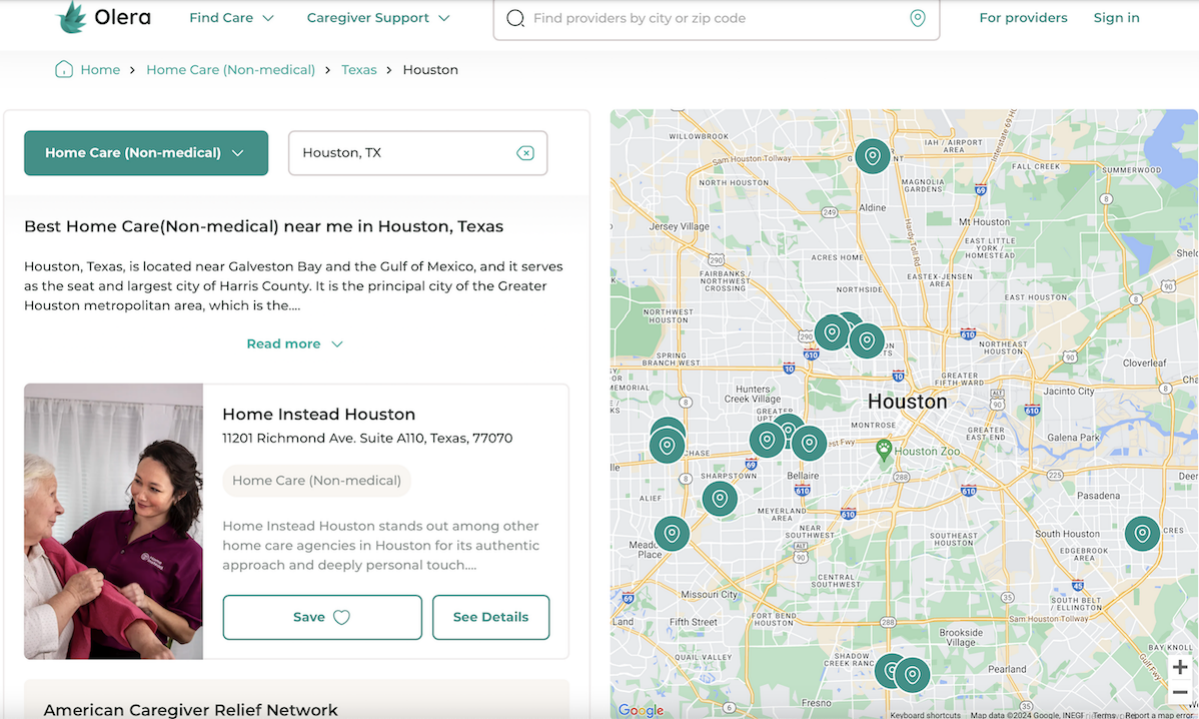
Information on local care on the Olera.care platform.

The platform is developed to be a free web-based service to assist caregivers in connecting them with educational and professional assistance. The educational materials (Figures 1 and 2) are created in collaboration with subject matter experts from local Area Agencies on Aging and Texas A&M University Center for Community Health and Aging. In addition, the National Institute on Aging, the American Association of Retired Persons, and the Texas Alzheimer’s Research and Care Consortium offer detailed informational materials on the legal and financial considerations for our targeted caregiver groups, which we frequently reference. Information on local care (ie, non–medical home care, assisted living, and nursing home) options (Figure 3) is gathered from public knowledge bases such as websites of Medicare and the websites of local Area Agencies on Aging (eg, Brazos Valley Area Agency on Aging, Harris County Area Agency on Aging, and Area Agency on Aging of the Capital Area). The primary objective of this step is to compile a comprehensive library of information on the functional, legal, financial, and estate planning aspects of AD/ADRD caregiving, along with a detailed list of care facilities in Texas.

The educational content gathered was organized after compilation. This process involved dividing the information into clear, manageable segments and classifying each segment into one of three categories: (1) legal, financial, or other services; (2) home care; and (3) senior communities. Once categorized, a logical decision tree was created to match each piece of information with specific combinations of caregiver characteristics provided by the user. The algorithm for this process was developed using Python (Python Software Foundation) due to its straightforward syntax, extensive open-source libraries, and capability for efficient data manipulation [41]. In the final website-based application, caregiver characteristics will serve as input criteria, including factors such as the dementia stage of the care recipient, the caregiver’s knowledge and skills, the availability of external resources, the care recipient’s insurance status, financial capacity for care, and family support presence. These criteria will be collected when users create their accounts for the first time, determining the personalized information and recommendations from each of the 3 categories that will be presented to the caregiver. The logic decision tree was ultimately converted into a user-friendly website-based platform for caregivers to access customized educational materials (eg, videos, articles) and relevant care options (eg, at-home care, older adults living arrangements, paying for care options, caregiver support). Google’s Firebase is employed for backend programming to enable seamless data synchronization, user authentication, and secure data management. The front-end user interface is built using Google’s Flutter software development kit, facilitating cross-platform development for Android, iOS, and web applications from a single codebase. This approach streamlines the development process and reduces long-term maintenance costs.

### Phase I: Mixed Methods Study—Needs Assessment and Pilot Testing

The phase I study is designed in a sequential mixed methods approach to understand the caregiving challenges and needs of caregivers and assess the platform’s usability with a pilot panel of AD/ADRD caregivers. The mixed-methodological design aims to enhance the depth and richness of data interpretation. The qualitative research serves as a need assessment, providing comprehensive information on the caregiving needs and expectations for the digital platform. Subsequently, the quantitative research evaluates the platform’s usability and usefulness for caregiving and identifies areas for platform enhancements.

The qualitative part involves semistructured interviews with caregivers, focusing on financial and legal challenges related to caregiving, unmet needs in financial management and legal planning, and expectations for a caregiving support platform. These interviews are recorded and transcribed for qualitative data analysis using thematic analysis in a framework approach [42]. Participants were also invited to complete a survey including questions about sociodemographic and caregiving characteristics, as well as questions on the usage and preferences for older adult care services.

A panel of dementia caregivers was invited to test the prototype web-based application by performing specific tasks, such as searching for financial and legal information. The study team is responsible for monitoring task completion, recording the status and time taken, and taking observation notes. After the prototype test, participants are invited to complete a usability survey to rate the perceived usefulness, ease of use, and overall rating of the web-based platform in supporting their caregiving needs. The usability of the platform was assessed using a modified MARS, which evaluates functionality, design, information quality, and engagement on a 5-point Likert scale [35]. The development goal is a MARS score of 3.6 or higher [43] among the pilot group of caregivers. The collected information will enable the development team to learn from the user feedback and plan for continuous product enhancement.

### Phase II: Usability Evaluation Study Among Diverse Racial and Ethnic Groups

The phase II study is a usability study that evaluates the acceptance of the platform among the 3 largest racial and ethnic groups in Texas (Black or African American, Hispanic or Latinx, and non-Hispanic Whites) from a diverse socioeconomic spectrum. Participants who are interested in the study will complete a short interest form consisting of 4 questions to provide contact information. Participants will then be contacted to complete an intake form via Qualtrics consisting of eligibility screening questions; an informed consent form; questions on their demographic, socioeconomic, and caregiving characteristics; and an assessment of care receipt’s dementia stage using a validated 8-item screening instrument, the Ascertain Dementia-8 [44]. Next, eligible participants can select either to explore the platform and complete the rest of the study on their own for greater flexibility or to schedule a guided session with 1 of the study staff over Zoom (Zoom Video Communications, Inc). With instructions from the study staff, participants will create an account on the Olera.care platform and generate a personalized caregiver checklist based on their needs by answering a few questions. After setting up their account, participants will explore and use the platform for 4 weeks. At the end of the 4 weeks, participants are invited to provide feedback by filling out an evaluation survey of the platform, the Technology Acceptance Survey, which was developed based on the TAM, a well-established framework for assessing the perceived usefulness and ease of use of information technologies [38,45].

### Participant Eligibility and Recruitment

To be eligible for phase I, participants must meet the following criteria: (1) be the primary unpaid caregiver for a person living with dementia, (2) provide a minimum of 10 hours of care per week to a person living with dementia who has not been institutionalized, (3) be an adult child, spouse, or family member of the person living with dementia, (4) have concerns about or perceive the need for additional information on financial management and legal planning for caregiving in Texas, and (5) have access to a smartphone or computer with internet connectivity. Paid formal caregivers will be excluded from this study. For the phase II study, the study participants must meet an additional criterion: be of Caucasian, African American, or Latino or Hispanic descent. Study participants are recruited in collaboration with the Center for Community Health and Aging at Texas A&M University and the Brazos Valley Area Agency on Aging. Web-based advertisements, emails, in-person presentations, and network recruitments are used to recruit participants. Potential participants will be invited to complete an eligibility assessment survey, and eligible participants will be invited to enroll in the study.

### Ethical Considerations

Human subject research approval (institutional review board [IRB] number: IRB2021-0943 D) was obtained from the Institutional Review Board at Texas A&M University, with phase I study approved on December 8, 2021, and phase II study approved on June 21, 2023. Electronic informed consents are obtained from study participants before study activities. Participants’ personal identifying information (eg, names, emails, phone numbers) was used solely for contacting purposes. We protected participants’ privacy and confidentiality by limiting access to IRB-approved team members, separating identifying information from deidentified study data, encrypting study information on Microsoft OneDrive, and deleting identifying information upon project completion. Participants in phase I received a US $25 e-gift card for completing both interviews and surveys, and phase II participants will receive up to US $50 e-gift card for completing the study.

### Sample Size Calculations

The phase 2 research hypothesis is that there will be a difference in the perceived usefulness and ease of use between different racial and ethnic groups. For the sample size calculation, we assumed a minimum power of 80% and a type I error rate of 5%. Due to the limited research on the influence of racial or ethnic factors on caregiving technology acceptance, we estimated the necessary sample size across a range of effect sizes (*f*²=0.02-0.35). Our analysis model will adjust for caregivers’ sociodemographic characteristics, caregiving duration, and the dementia stage of the care recipient. Depending on the effect sizes, the required sample size varies from approximately 40 to 550 participants [46]. Previous studies have shown low retention rates among informal caregivers for individuals with AD/ADRD, around 50% [47,48], and even lower retention rates among Hispanic or Latinx caregivers [47]. By following recommended recruitment and retention strategies and drawing on the recruitment experience of Texas A&M Center for Community Health and Aging in various community and clinical projects, we estimate that a minimum total sample size of 300 for enrollment surveys (100 per group) with 150 for conclusion surveys (50 per group) is necessary. This minimum sample size of 150 caregivers is feasible and can detect a small effect size of 0.09.

### Data Analysis

For phase I data, thematic analysis of qualitative interview transcript data was conducted using the framework method [42,49]. The framework method consists of several essential steps: transcribing interviews, familiarizing oneself with the interview material, coding, developing an analytical framework, applying this framework, charting data into the framework matrix, and interpreting the data [42]. In addition, descriptive analyses of quantitative survey responses will be conducted to describe the sociodemographic and caregiving characteristics of the participants, their usage and preference for care services, and their evaluations of the platform’s functionality, design, information quality, and engagement.

For phase II survey data, descriptive statistics (mean and SD or frequency and percentage) will be used to describe the characteristics of study participants and their perceived usefulness and ease of use of Olera.care digital platform. First, using the Technology Acceptance Survey data, separate multivariable regression models will be used to examine any differences in each key outcome (perceived usefulness and ease of use of Olera digital platform) by racial or ethnic characteristics and socioeconomic status (eg, education and income level). The regression model will be adjusted for known factors that influence technology adoption (eg, age, sex, and care recipients’ dementia stage).

## Results

### Overview

The study received funding from the National Institute on Aging on September 3, 2021. Ethical approval for phase I was obtained from the Texas A&M University Institutional Review Board on December 8, 2021, with data collection starting on January 1, 2022, and concluding on May 31, 2022. Phase I results were published on September 5, 2023, and April 17, 2024, respectively. On June 21, 2023, ethical approval for human subjects for phase II was granted, and participant recruitment began on July 1, 2023, and is anticipated to end by December 31, 2024. We expect to publish our phase II results by June 30, 2025. The Olera.care platform has seen promising growth since its official launch in 2023, now with more than 350 daily logins and a steady increase in organic traffic from August 6, 2023, to June 1, 2024 (Multimedia Appendix 2). In addition, our Facebook “Health Aging Community” has more than 11,000 followers by June 1, 2024. The goal is to expedite this organic growth of our website and social media platforms in a way that requires no paid advertisements and is sustained only by quality content on the website attracting visitors (organic traffic). Our goal is to reach more than 30,000 caregivers daily by June 30, 2027. This will be achieved through continued investment in quality content, strategic partnerships, and community engagement.

### Platform Description and Functionality

The primary function of the Olera.care platform is to provide personalized recommendations for learning materials, including articles and videos, tailored to caregivers’ specific needs. The platform also offers recommended listings of local service providers across various domains such as legal and financial advisors (eg, older adults law attorneys, certified financial planners), home care providers, senior living options, and public support services such as Meals on Wheels and Area Agencies on Aging. This personalized approach aims to simplify the web-based research process for caregivers, providing a guided experience akin to expert advice.

**Figure 4:**
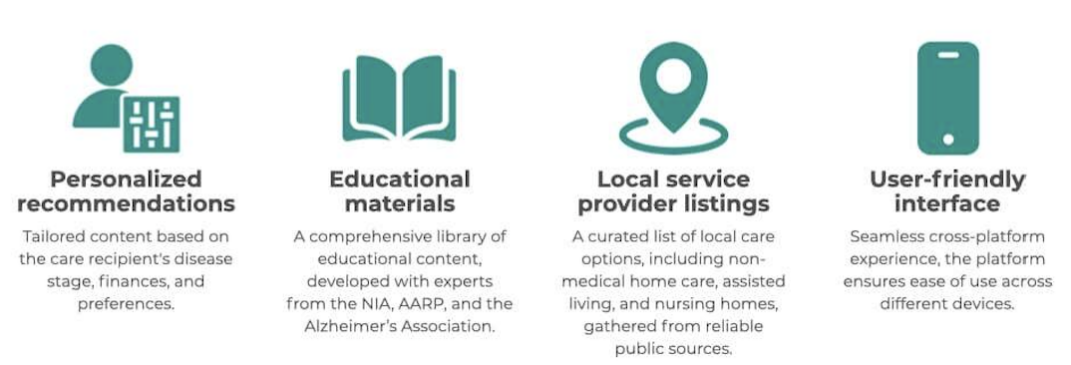
Key features of the Olera.care platform. AARP: American Association of Retired Persons; NIA: National Institute on Aging.

Key features of the Olera.care (Figure 4) include the following:

1. *Personalized recommendations*: the DCPA customizes content based on the care recipient’s disease stage, financial circumstances, and care preferences. This ensures that caregivers receive relevant, contextually appropriate information and resources.
2. *Educational materials*: The platform hosts a comprehensive library of educational content created in collaboration with experts from various organizations, including the National Institute on Aging, the American Association of Retired Persons, and the Alzheimer’s Association.
3. *Service provider listings*: Caregivers can access a curated list of local care options, including non–medical home care, assisted living, and nursing homes, gathered from reliable public sources.
4. *User-friendly interface*: developed using Ruby on Rails web application framework for a seamless cross-platform experience, the platform ensures ease of use across different devices.

### Marketing and User Growth Strategy

Since its launch, the Olera platform has focused on building organic traffic through several key strategies:

#### Community Building

The “Healthy Aging Community,” a web-based space hosted on Facebook, for seniors and caregivers, has become a significant marketing channel, with more than 11,000 registered members. This community fosters engagement and word-of-mouth promotion, driving new users to the platform.

#### Content Marketing and Search Engine Optimization

The platform’s Educational Material and Caregiver Resource repository features more than 20,000 indexed pages of high-quality content (Multimedia Appendix 3). This content attracts organic traffic from users searching for relevant caregiving information, supported by digital public relations efforts and outreach to journalists, bloggers, and podcast hosts.

#### Strategic Partnerships:

Collaborations with organizations such as the Alzheimer’s Association and Texas A&M Center for Community Health and Aging have expanded the platform’s reach. These partnerships are crucial for user growth, with plans to engage more caregivers through connections with local Area Agencies on Aging and older adult centers.

#### Promotion to Service Providers

Marketing efforts also target service providers in the older adult care industry. Promotions for new providers and participation in industry conferences help build a robust network of providers on the platform. When families like a provider, a lead can be generated for that organization when we forward their contact information. Weekly leads are directly related to the volume of organic visits we get from caregivers using the platform (Multimedia Appendix 4).

### Study Progress by June 30, 2024

Phase I study data collection started on January 1, 2022, and ended on May 31, 2022. For participants, 822 respondents filled out prescreening surveys and 150 (18.2%) of them were qualified. Approximately 20% (30/150) of the eligible respondents participated in the in-depth interviews and completed the survey. The preliminary results of the phase I study were disseminated in a few regional or national conferences (ie, 2024 annual meeting of American Academy of Health Behavior; 2024 Texas Alzheimer’s Research and Care Consortium Symposium; 2023 Healthy Aging and Dementia Research Symposium; and 2023 annual meeting of American Association for Geriatric Psychiatry), and formal results were published on September 5, 2023 [8] and April 17, 2024 [50].

Phase II study commenced on July 1, 2023, and we aim to complete recruitment and data collection by the end of December 31, 2024, and conduct analysis and report our phase II evaluation results by June 30, 2025. By thoroughly evaluating the Olera.care digital platform across diverse sociodemographic groups, we aim to ensure that the platform is both effective and user-friendly for a broad audience. This evaluation will provide critical insights into the specific needs and preferences of different caregiver populations, enabling us to tailor the platform more precisely.

## Discussion

### Principal Findings

This study presents an iterative approach to developing, refining, and evaluating a novel caregiver assistive technology. Current literature regarding the efficacy and acceptance of predeveloped caregiver interventions, while able to provide context regarding caregiver needs and future directions, often overlooks user feedback during the developmental phase. Using a build-measure-learn approach, the development of the Olera.care platform incorporates caregiver opinions and thoughts throughout the process. In the phase I study, we used a mixed methods approach to obtain comprehensive feedback in both qualitative and quantitative measures and examine the quality of the developed platform using the validated MARS tool. In the phase II study, we evaluated the acceptance of the technology among 3 major racial and ethnic groups using the TAM framework. This enables a comparison of measurements in perceived ease of use and usefulness, similar to another study that used the TAM framework to evaluate the acceptability of a care coordination platform [51].

A significant difference between our product and recently developed caregiver platforms is the multifaceted nature of the Olera.care platform. While many caregiving assistive technologies aim to decrease caregiver burden and strain, they mainly focus on only 1 or 2 aspects of the caregiving experience, thus limiting their use. For example, while the end goal is the same, these technologies often target specific aspects such as caregiver management [51-54], clinical reasoning [55], caregiver education and training [56,57], or caregiver mental well-being [58]. Olera.care platform serves functions in all 4 of the identified components and thus is able to serve as a simplified hub, capable of assisting caregivers in all needed aspects. For these reasons, Olera.care is a more rounded and comprehensive intervention than existing platforms.

### Strengths and Limitations

One of the key strengths of the study is the build-measure-learn process. This iterative approach ensures that caregiver feedback is incorporated throughout the development phase, leading to a more user-centric design. By continuously refining the platform based on user input, the Olera.care platform is better tailored to meet the actual needs and preferences of caregivers, enhancing its usability and effectiveness. Another significant strength is the mixed methods approach in phase I, which allows for the collection of both comprehensive qualitative data on caregiving needs and expectations, as well as quantitative measures of usability and usefulness using the validated tool, MARS. The majority of similar studies have evaluated the acceptance of an intervention using only qualitative measures as opposed to quantitative [52,53,55]. Other studies have demonstrated the use of a mixed methods approach and MARS in broadening the caregiver perspective [54,56,59,60]. While app quality can be assessed in a multitude of ways, it is important to consider the MARS during developmental testing to prevent poor quality, especially considering that the average caregiving app was found to be “of [minimal] acceptable quality” that is “likely to be insufficient to meet care partner needs” [61]. Furthermore, this study stands out by investigating the acceptance of the technology among diverse ethnic and racial groups of caregivers in phase II. It is important to note the necessity of a diverse study population, considering the aforementioned burden disparities in racial and ethnic groups. Limitations in similar studies often arise from a lack of generalizability and consideration of a broadly representative sample [56,57]. Often, the unintentional focus on non-Hispanic White populations materializes in a lack of culturally tailored interventions [62]. This study will help bridge this gap by focusing on the needs and feedback across racial or ethnic populations.

The study has several limitations typical to research in this field of study. Recruiting racially and ethnically diverse caregiver participants, specifically from the 3 largest groups in Texas: non-Hispanic White, Hispanic or Latinx, and African American individuals requires intense efforts. Despite significant efforts to collaborate with communities and local agencies for recruitment, achieving a truly representative sample remained challenging. In addition, we were unable to include other racial or ethnic groups such as Asian American, Pacific Islander, Indian American, and Multiracial American. This omission is noteworthy as it potentially excludes populations that could offer important insights. However, the decision to focus on the 3 major groups was based on examining differences across these groups and considering the feasibility of recruiting caregivers in Texas. In addition, while our sample was racially diverse, it may not have been socioeconomically diverse and representative of all residents in Texas and the United States. Therefore, it is important to compare our participants’ characteristics with Texas caregiver profiles and national caregiver profiles to determine the representativeness of our recruited sample. Despite measuring household income levels and self-reported financial situations, unmeasured factors may influence the acceptance and perceived usefulness of the platform. For instance, health literacy and technology literacy can significantly impact the access and use of web-based caregiving resources [63,64]. Another limitation is the lack of longitudinal testing to observe changes in perceived usefulness over time. This gap means we are unable to assess how perceived acceptance and ease of use might evolve as caregivers continue to use the platform and their caregiving responsibilities evolve. Longitudinal studies are essential to understanding the sustained impact and usability of interventions over time. This underscores the need for a longitudinal study design and a more comprehensive assessment of caregiver characteristics in our future studies. By addressing these limitations in future research, we can gain a deeper understanding of the long-term effectiveness and broad applicability of the platform across various demographic and socioeconomic groups.

### Conclusions

This 2-phase study focuses on caregiver-based iterative development and evaluation of the quality and usability of the platform among caregivers. The development of our platform incorporates the needs and opinions of end users throughout the entire process to ensure the creation of an effective product. The platform is rigorously assessed regarding overall app quality and acceptability using validated tools in a pilot group and across diverse caregivers, respectively. The phase I results demonstrate that digital platforms, especially those offering personalized and comprehensive support, hold significant promise for supporting family caregivers of people living with dementia. By the end of the study, we expect to deliver a highly accessible digital platform designed to assist dementia caregivers in managing financial and legal challenges by linking them to personalized and contextually relevant information and resources in Texas. If the Olera.care platform demonstrates the usefulness and ease of use, we plan to collaborate with caregiving organizations to expand it nationally, addressing the needs of the growing population of dementia caregivers.

## Appendix

Multimedia Appendix 1 NIH peer review summary statement for original proposal.

Multimedia Appendix 2 Number of visitors who found Olera.care website from free traffic of search engines by June 1, 2024.

Multimedia Appendix 3 Number of pages indexed by Google by June 1, 2024.

Multimedia Appendix 4 Number of client requests by June 1, 2024.

## Abbreviations

AD: Alzheimer disease
AD/ADRD: Alzheimer disease and Alzheimer disease–related dementia
AI: artificial intelligence
DCPA: Dementia Care Personalization Algorithm
IRB: institutional review board
LLM: large language model
MARS: Mobile Application Rating Scale
TAM: Technology Acceptance Model

## Data Availability

The data sets generated and analyzed during this study are not publicly available due to protection of study participants’ privacy, but de-identified data sets are available from the corresponding author on reasonable request.
