# Supplementary material for: Development and Evaluation of a Web-Based Platform for Personalized Educational and Professional Assistance for Dementia Caregivers: Proposal for a Mixed Methods Study
Source: JMIR Res Protoc. 2024 Aug 7;13:e64127. doi: 10.2196/64127 (PMC11339571; doi:10.2196/64127)
Supplement: Multimedia Appendix 1 [file resprot_v13i1e64127_app1.pdf]

SUMMARY STATEMENT

(Privileged Communication)

PROGRAM CONTACT:
Dana Plude
301-435-2309
dana.plude@nih.gov

Release Date: 03/07/2021
Revised Date:

Application Number: 1 R44 AG074116-01

Principal Investigator

FALOHUN, TOKUNBO

Applicant Organization: OLERA INC.

Review Group: ZRG1 RPHB-W (15)
Center for Scientific Review Special Emphasis Panel
RFA-AG-21-025: Training and Education for AD/ADRD Caregivers on Financial Management and Legal Planning

Meeting Date: 02/25/2021
Council: MAY 2021
Requested Start: 07/01/2021
RFA/PA: AG21-025
PCC: 2BCOGDP

Project Title: Olera - Online Platform to Increase Access to Personalized Educational and Professional Assistance for AD/ADRD Caregivers
SRG Action: Impact Score:20
Next Steps: Visit https://grants.nih.gov/grants/next_steps.htm
Human Subjects: X3-Human subjects involved - Exemption #3 designated
Animal Subjects: 10-No live vertebrate animals involved for competing appl.
Gender: 1A-Both genders, scientifically acceptable
Minority: 1A-Minorities and non-minorities, scientifically acceptable
Age: 3A-No children included, scientifically acceptable

Project Year	Direct Costs Requested	Estimated Total Cost
1	221,232	280,423
2	736,322	933,326
3	736,322	933,326
TOTAL	1,693,876	2,147,074

ADMINISTRATIVE BUDGET NOTE: The budget shown is the requested budget and has not been adjusted to reflect any recommendations made by reviewers. If an award is planned, the costs will be calculated by Institute grants management staff based on the recommendations outlined below in the COMMITTEE BUDGET RECOMMENDATIONS section.

1R44AG074116-01 Falohun, Tokunbo

RESUME AND SUMMARY OF DISCUSSION: This SBIR Fast Track application proposes to develop a digital platform OLERA (a mobile phone and website app), which incorporates a novel dementia care personalization algorithm to provide educational materials and a resource library to assist caregivers of people living with dementia (PLWD) with legal, financial and estate planning aspects of caregiving. The project is built on rigorous prior research and a prototype that has received positive usability feedback. The personalization and geolocation algorithms are highly innovative, as is its culturally sensitive design. The research strategy includes rigorous and detailed methods, with clear milestones to progress from Phase 1 to Phase 2. The feasibility is clear, as are qualitative and quantitative analyses. Alternative approaches are discussed. The commercialization plan is well-developed and realistic. The research team has complementary expertise. The review panel has high confidence that this project will be successful. There were only minor concerns. There was a lack of consideration of caregivers who might not have mobile phone or Internet. The commercialization plan had some vulnerabilities in terms of delivering projected revenue streams. There was not a full discussion of how privacy of individual data would be protected. Overall, this is a very significant and viable project, and it could have a major impact on the field.

DESCRIPTION (provided by applicant): There are currently about 5.8 million older adults living with Alzheimer’s dementia, and the numbers are only expected to grow in the coming decades. The majority of care for persons living with dementia (PLWD) is provided by family members who lack formal training in caregiving and thus need education, information, and assistance. While an array of technology solutions for caregiving has been suggested to meet these needs, the adoption rate has been low, potentially due to poor usability (e.g., difficult to use) and lack of contextual relevancy. The long-term objective of this proposed research is to develop a technology-based solution that integrates web and mobile platforms to link dementia caregivers with personalized information and resources that are easily accessible by populations across the socioeconomic spectrum. The proposed study will focus on information and educational materials on financial management and legal planning. Such information will be helpful for caregivers experiencing difficulties due to their immediate caregiving responsibilities, as well as those transitioning in their roles and responsibilities as the PLWD’s health condition progresses. This collaborative project, involving industrial, academic, and community partners, is oriented around two specific aims: (Phase 1 Specific Aim 1) Development of the digital platform based on a novel Dementia Care Personalization Algorithm and initial feasibility assessment. Framed around an interactive Design Thinking product design methodology, phase 1 consists of the iterative process: Build, Measure, and Learn. A pilot group of 25 dementia caregivers will be recruited to serve as a user feedback panel. Our milestone for moving to Phase 2 will be assurance of the feasibility of the digital platform assessed using metrics from the adapted Mobile Application Rating Scale (MARS) and Technology Adoption Model (TAM). (Phase 2 Specific Aim 2) Evaluation of the acceptance of the developed digital platform across racial/ethnic populations. Phase 2 is a prospective usability study to evaluate the acceptance of the proposed digital platform across three different racial/ethnic populations of dementia caregivers in Texas (N = 300; n = 100 African Americans, n = 100 Hispanic/Latinx, and n = 100 non-Hispanic Whites). The prospective feature of the study design will enable the enrolled participants to explore and use the developed digital platform for about 4 weeks to provide multiple assessments of the perceived usefulness, ease of use, and value. At the completion of the two specific aims, we expect to have developed a widely accessible digital platform tailored to assist dementia caregivers in navigating through financial and legal challenges by linking caregivers to personalized and contextually relevant information and resources in the Texas area. If successful, the proposed commercialization plan anticipates further deployment of the developed digital platform by working with professional caregiving organizations to scale and sustain the platform to meet the needs of the growing population of dementia caregivers.

PUBLIC HEALTH RELEVANCE: This is a three year fast-track SBIR application with phase I scheduled for 12 months and phase II for 24 months. The timeline in the research plan details tasks by phase (reflecting specific aims) and further by quarter. All milestones for phase 1 must be met before moving to phase 2. The project will develop the innovative digital platform incorporating a novel Dementia Care Personalization Algorithm and Educational Materials and Caregiver Resources library to assist caregivers of people living with dementia (PLWD). The product of this project will be commercially viable and poised for distribution across the US where it will help millions of caregivers of PLWD.

CRITIQUE 1

Significance: 2
Investigator(s): 2
Innovation: 2
Approach: 1
Environment: 5

Overall Impact: The applicant proposes fast track development of a cross-platform mobile phone and website app using a novel dementia care personalization algorithm (DCPA) to provide educational materials and a vetted resource library (EMCR) for professional assistance with legal, financial, and estate planning aspects of caregiving, tailored to diverse racial and ethnic family caregivers of those with AD/ADRD in the State of Texas. This is offered at no cost to the end user. Significance of the problem and appropriateness of the intervention are supported with past rigorous research and a funded study of a prototype. The commercialization plan is comprehensive. The innovation of the app is in the personalization and geolocation algorithms and its culturally sensitive design. The research strategy demonstrates scientific rigor with the Phase II approach based on the results of Phase I. The investigative team is strong in digital technology and design, dementia/caregiver issues, cultural issues, and curriculum development. Because the emphasis is on legal, financial, and estate planning for this population, the team would be strengthened with the addition of experts in elder law and finance. It is not clear in the application in what environment the application will be developed. There are minor concerns that there is no training manual/users guide and that users might be hesitant to divulge personal information to the app, negating its use. These do not dampen enthusiasm for the application.

1. Significance:

Strengths

- Utility of the proposed product is supported with statistical data and past rigorous scientific research, including data on the use of technology by African American and Hispanic/Latinx caregivers.
- A prototype has received positive user feedback from 50 caregivers and stakeholders through funding from the National Science Foundation Innovation Corps site program.
- Product is proposed to support not only caregivers, but also serve as an access point to caregivers for professional organizations that support the app.
- Commercialization potential is supported with preliminary market data, evaluation of three existing products, and presentation of potential revenue streams. Software codes will be copyright protected.

Weaknesses

- Product development and testing in this Fast Track will include only caregiver resources in the State of Texas (but with the intent to expand to the entire US).
- Preliminary market analysis is based on senior housing and home care resources data only, not the broader network of professional caregiver assistance organizations.

2. Investigator(s):

Strengths

- PI Falohun has industry experience as a biomedical engineer and entrepreneurial experience as the founder of an interdisciplinary technology organization in his graduate program, and co-founder of his current startup company, Olera, to develop and commercialize digital solutions to medical problems. He and his company have no history of commercialization, but are supported by a team with those skills.
- Co-Investigator, DuBose, company VP, will receive MBA and MD degrees by 2022 and manages the commercialization strategies for the company under the mentorship of the Director of Technology Development at Texas A&M.
- PI and Co-PI have experience in their graduate programs managing multiple teams.
- The investigative team for Texas A&M brings strong credentials in research and publication. Dr. Alonzo, PharmD, has R&D expertise in healthcare technology solutions for underserved populations, though none are specific to dementia.
- Dr. Ory, a former Program Director at the NIA and founding Director, Center for Population Health and Aging, School of Public Health, Texas A&M has an extensive history in dementia care and caregiver burden among diverse populations, and in establishing strategic partnerships and initiatives. She is PI on five current, variously funded studies related to technology support for persons with dementia and their caregivers.
- Dr. Pena-Purcell works extensively in developing evidence-based educational content for Latinx and African American minority health and in cultural competency – again, not AD/ADRD specific.
- Dr. Lee (Advisory Board) has translational research experience with technology-based interventions among caregivers of PWD.
- Dr. Opperman's (Advisory Board) has expertise in collaborating with small businesses to bring products to market. None are related to dementia.
- The Olera Advisory Board also includes expertise in taxes and law, and a safety net medical clinic director with direct experience supporting those with dementia and their caregivers.

Weaknesses

- To date, Olera has no history of bringing a product to market.
- The team would be strengthened with members or advisors with specific expertise in elder law and financial planning.

3. Innovation:

Strengths

- Product's mobile app and website use logic-decision trees and geolocation to personalize both education and resource acquisition.
- Product employs user feedback allowing the algorithm to learn and adapt.
- Product has a dynamic interface that promotes active learning.
- App includes psycho-educational tools to support caregivers.
- Designed to be culturally and economically relevant for African American and Hispanic/Latinx families as well as for the non-Hispanic White population.
- Prototype interface is visually pleasant.
- Design is based, in part, on 50 prior prototype assessment interviews and data analysis conducted with dementia caregivers and key stakeholders.

Weaknesses

- There is no training guide for those who are technologically naïve, however it is suggested that future iterations may consider voice assistance technology.
- There is the potential that users will be reluctant to divulge personal data within the app.

4. Approach:

Strengths

- Phase I tasks and milestones are clearly outlined using the iterative Build-Measure-Learn model with feedback from 25 caregivers using both qualitative data and the validated Mobile Application Rating Scale (MARS) with an identified significance level, to establish initial feasibility/usability.
- Inclusion/exclusion criteria for the research strategy in each Phase are clearly articulated to include adult children, spouses or other family members only.
- Study participant recruitment for Phase II includes Statewide and local agencies that have partnership histories with The Center for Population Health and Aging, uses various methods of outreach, and is based on local and national demographics.
- The Phase II research strategy builds on Phase I. The research protocol tests the product over a 4-week period with African American, Hispanic/Latinx, and non-Hispanic Whites. Protocol involves qualitative/quantitative measures to establish usability within each group. Power calculations and hypothesis testing are described.
- Potential problems and alternative approaches are discussed.
- Study timeline is reasonable for the tasks in each Phase.
- Major roles of each member of the investigative team are described.

Weaknesses

- Risks and benefits to human subjects participating in the research strategy for Phases I and II are not identified.

5. Environment:

Strengths

- Olera's focus is on development and commercialization of innovative digital medical products with a current focus on dementia care. The company has a strong advisory board and close times to Texas A&M facilities and faculty.
- Resources and faculty at Texas A&M and the Center for Population Health and Aging have commitments in writing to support the project.

Weaknesses

- It is unclear where the digital application will be developed and how development software will be accessed. There is no budget line item for equipment and no description of a location.

Study Timeline:

Strengths

- None noted by reviewer

Weaknesses

- None noted by reviewer

Fast Track (Type 1 R42 and Type 1 R44 applications):

Acceptable

- Proof of Concept (Phase I) and acceptance/usability (Phase II) are clearly articulated.

Protections for Human Subjects:

Acceptable Risks and Adequate Protections

- Risks and protections need to be articulated. Though not specified in this application, it will clearly be required in the IRB applications.

Data and Safety Monitoring Plan (Applicable for Clinical Trials Only):

Not Applicable (No Clinical Trials)

Inclusion Plans:

- Sex/Gender: Distribution justified scientifically
- Race/Ethnicity: Distribution justified scientifically
- For NIH-Defined Phase III trials, Plans for valid design and analysis: Not applicable
- Inclusion/Exclusion Based on Age:
- No one under the age of 18 is included. Sex/gender distributions are based on national demographics within the specified racial/ethnic groups (Phase II)

Vertebrate Animals:

Not Applicable (No Vertebrate Animals)

Biohazards:

Not Applicable (No Biohazards)

Resource Sharing Plans:

Acceptable

Budget and Period of Support:

Recommend as Requested

CRITIQUE 2

Significance: 2
Investigator(s): 2
Innovation: 2
Approach: 2
Environment: 2

Overall Impact: This is a novel approach to both aggregating but also customizing data on legal and financial decisions and resources for ADRD caregivers using a web-based platform, mobile app for on demand convenience and proprietary algorithm that customizes the recommendations. The collaborative support from Texas A&M University, including ADRD clinical experts and an existing Advisory Board of local community organizations for the platform, demonstrate viability and probably validity of the proposal. The Commercialization plan in Phase III is based solely on advertising and client referral revenues which will be based on achieving end-user goals in Phase II and partnership outreach in Phase I.

1. Significance:

Strengths

- The personalization aspect of the proposal, especially for cultural, racial, socioeconomic and demographic filters, is novel in this space, particularly for legal and financial planning purposes.
- If Specific Aims are achieved in each Phase, the proposal provides a unique solution to existing challenges for ADRD caregivers and will create a more educated, empowered and less stressed caregiving population that has the downstream effect of potentially better patient-centered care and more financial stability for families.
- While not specifically called out as potential impact in the proposal, the information about the “Daily Caregiving Guide” customized to each user has strong potential for continued user engagement and psychosocial benefits of making caregivers feel part of a community rather than having the platform be just an information and education source.
- Specific milestones for each Phase are indicated with responsive assessment and analysis for moving the project forward to achieve Specific Aims.

Weaknesses

- The Commercialization Plan (Phase III) has potential based on advertising revenues models and customer referral programs. The vulnerability in the plan is that ad revenues will be based on users (who get free access), so fulfilling the proposed goals in Phase II is critical (4 million

users by 2024). Also, referral programs differ per organization with some only paying for secured placements instead of simple leads, others paying for warm leads versus cold leads. And, if the platform is truly comprehensive, then recommendations to caregivers will need to also include leads from organizations who do not pay for referrals – this would differentiate from the business models established by noted competitors such as A Place for Mom and Caring.com.

- While most of the educational material to be delivered via the platform is sourced to other expert organizations, it was not apparent in the budget ifs there is a line item for licensing such materials from certain groups (for instance the Alzheimer's Association has a licensing fee for all of its materials usage).

2. Investigator(s):

Strengths

- The PIs have complementary strengths that aid in the viability of the project. Particularly, Dr. Ory's expertise and passion for ADRD caregiver support is matched by Mr. Falohun as the digital product and tech expert supplemented by Dr. Joy Alzono in the Pharmacy Dept. who has experience in deployment of software tools in patient populations.
- As well the collaboration with Texas A&M provides foundational support (including IP legal, regulatory and IRB guidelines) and access to an existing cohort of potential ADRD caregivers via health center and patient populations to participate in the validation stages.
- Eliciting participation with local community and government service organizations, including the Advisory Committee (AAAs, NAELA, Alzheimer's Association) in Phase I research and discovery is a key strength of the proposal.

Weaknesses

- To drive the user numbers needed to move to Commercialization in Phase III, strong marketing and community partner outreach is needed yet a marketing strategist is not indicated until this last Phase. This may impact some of the projected revenue goals in the first 12 months of the official launch in Phase III as the project ramps up on engagement.

3. Innovation:

Strengths

- The two biggest strengths of the proposal are the specialized algorithm (DCPA dementia caregiver personalization algorithm) to be developed to deliver the customization/personalization of information to individual caregivers; and the dedicated to delivering information in a culturally, racially, socioeconomically sensitive ways. By crossing the different sub-cohorts of caregivers (which also includes demographics, especially age of caregiver), this level of personalization appears to be novel and impactful in the ADRD caregiver support space.
- The proposal also has generalizability to deploy the concept to a broader cohort of caregivers beyond ADRD. Scalability will be predicated upon a similar approach used in Phase I for each local county/state to engage with local organizations and assess varying caregiver needs by geographics along with all the other data. This will be especially critical for rural and tribal areas where resources are more limited.

Weaknesses

- It is unclear in the proposal if the specialized algorithm is based on the more simple process through decision mode ("if this, then this") or if it will use machine learning or AI to continually customize the data for caregivers.
- The Commercialization Plan has some potential vulnerabilities in terms of delivering projected revenue streams. The proposal indicates other potential referral clients beyond senior living, nursing homes and home care such as elder law attorneys, financial planners but does not indicate health plans, individual care managers, benefit managers and other potential sources.

4. Approach:

Strengths

- The proposal is comprehensive in its outline of each Phase of the project leading to Commercialization in Phase III. Milestones and assessment, analysis are well-woven into the planning to achieve feasibility and proof of concept. Especially the transition from Phase I to Phase II indicates analysis of the concept based on Phase I findings.

Weaknesses

- It is unclear in the proposal how privacy of individual data will be secured, stored and protected.
- The proposal relies on content provided by external expert sources (e.g., Alzheimer's Association). However, it does not indicate how to develop original materials if there are gaps in the outsourced materials.
- While the empowerment of caregivers through education and information is solid in the proposal, what is less clear is how the caregivers will be advised on the next step for actionable planning (creation of legal documents, in-person financial planning). Referral sources are referenced as part of the Commercialization Plan but the question is will the platform only recommend paying referral clients (like the Caring.com or A Place for Mom competitors' model) or will recommendations be based on all sources and resources regardless of the revenue connection?

5. Environment:

Strengths

- The academic medical center environment and institutional support will aid in the viability of the proposal and success of Phase I and II.
- The collaboration with community-based organizations (Alzheimer's Association and NAELA) and local government services (AAAs) also strengthens the success of the project as it moves through Phase I – III.
- The mission to deliver a diverse caregiver cohort (racially, culturally, demographically, socioeconomically) to drive personalization in a comprehensive way also makes the proposal a novel concept and greater engagement potential for Commercialization.

Weaknesses

- None for this area.

Study Timeline:

Strengths

- None noted by reviewer

Weaknesses

- None noted by reviewer

Fast Track (Type 1 R42 and Type 1 R44 applications):

- None noted by reviewer

Protections for Human Subjects

- None noted by reviewer

Data and Safety Monitoring Plan (Applicable for Clinical Trials Only):

Inclusion Plans:

- Sex/Gender:
- Race/Ethnicity:
- For NIH-Defined Phase III trials, Plans for valid design and analysis:
- Inclusion/Exclusion Based on Age:

Vertebrate Animals:

- None noted by reviewer

Biohazards:

- None noted by reviewer

Budget and Period of Support:
Recommend as Requested

CRITIQUE 3

Significance: 2
Investigator(s): 1
Innovation: 1
Approach: 2
Environment: 1

Overall Impact: This project aims to develop a technology-based solution that integrates web and mobile platforms to link dementia caregivers with personalized financial and legal planning information and resources that are easily accessible by populations across the socioeconomic spectrum. This technology would have a major impact on AD/ADRD caregivers by providing an easy-to-use, customized resource to aid financial and legal planning and reducing caregiver burden and time

demands. The proposal is well-written; includes rigorous, well-described and detailed methods; rigorous preliminary feasibility data; an excellent complementary team and environment; is innovative in use of both individual and geolocation information; and presents a well-developed and realistic commercialization plan. Weakness are few and minor. The project is judged to be of high impact.

1. Significance:

Strengths

- Addresses highly important topic of AD/ADRD needs for tailored interventions and resources on financial management and legal planning.
- Rigorous prior research on prototype / feasibility.
- Focus on growing populations of African American and Hispanic AD/ADRD caregivers.
- Incorporation of transitions as PLWD condition worsens.
- Clearly stated and justified Phase I milestones.
- Well-developed and realistic commercialization plan.

Weaknesses

- Does not address AD/ADRD caregivers without smartphones / internet access.

2. Investigator(s):

Strengths

- Excellent team with complementary expertise / skills in engineering; AD/ADRD caregiving; telehealth; culturally appropriate interventions; chronic disease management; and clinical research.

Weaknesses

- None noted by reviewer.

3. Innovation:

Strengths

- Use of both individual context / needs and geolocation information in algorithm.
- Continuous feedback to improve tailoring of intervention.
- Attempt to provide culturally relevant tailoring, which addresses both practical and scientific issues.

Weaknesses

- None noted by reviewer.

4. Approach:

Strengths

- Systematic use of the Build, Measure, Learn approach.
- Detailed methodology plan description, including case study.

- Systematic and strategic (at 3 time points) user feedback panel and TAM model to assess feasibility
- Well-designed, rigorous Phase II prospective usability study.
- Well-developed statistical analysis plan.

Weaknesses

- Unclear how Phase II usability respondents will be able to evaluate the new platform before use.
- No discussion of potential attrition / dropout bias analysis, given the possibility of losing up to half of the participants.
- Project does not address those AD/ADRD caregivers without access to smartphones / internet access.

5. Environment:

Strengths

- Excellent infrastructure and prior experience at both Olera and Texas A&M.

Weaknesses

- None noted by reviewer.

Study Timeline:

Strengths

- None noted by reviewer.

Weaknesses

- None noted by reviewer.

Fast Track (Type 1 R42 and Type 1 R44 applications):

Acceptable

- Clear Phase I milestones; high likelihood of commercialization.

Protections for Human Subjects:

Acceptable Risks and Adequate Protections

- Acceptable human subjects.

Data and Safety Monitoring Plan (Applicable for Clinical Trials Only):

Inclusion Plans:

- Sex/Gender: Distribution justified scientifically
- Race/Ethnicity: Distribution justified scientifically
- For NIH-Defined Phase III trials, Plans for valid design and analysis:
- Inclusion/Exclusion Based on Age: Distribution justified scientifically

- Acceptable human subjects.

Vertebrate Animals:
Not Applicable (No Vertebrate Animals)

Biohazards:
Not Applicable (No Biohazards)

Budget and Period of Support:
Recommend as Requested

THE FOLLOWING SECTIONS WERE PREPARED BY THE SCIENTIFIC REVIEW OFFICER TO SUMMARIZE THE OUTCOME OF DISCUSSIONS OF THE REVIEW COMMITTEE, OR REVIEWERS' WRITTEN CRITIQUES, ON THE FOLLOWING ISSUES:

PROTECTION OF HUMAN SUBJECTS: ACCEPTABLE

INCLUSION OF WOMEN PLAN: ACCEPTABLE

INCLUSION OF MINORITIES PLAN: ACCEPTABLE

INCLUSION ACROSS THE LIFESPAN: ACCEPTABLE

COMMITTEE BUDGET RECOMMENDATIONS: The budget was recommended as requested.

Footnotes for 1 R44 AG074116-01; PI Name: Falohun, Tokunbo

NIH has modified its policy regarding the receipt of resubmissions (amended applications).See Guide Notice NOT-OD-18-197 at https://grants.nih.gov/grants/guide/notice-files/NOT-OD-18-197.html. The impact/priority score is calculated after discussion of an application by averaging the overall scores (1-9) given by all voting reviewers on the committee and multiplying by 10. The criterion scores are submitted prior to the meeting by the individual reviewers assigned to an application, and are not discussed specifically at the review meeting or calculated into the overall impact score. Some applications also receive a percentile ranking. For details on the review process, see http://grants.nih.gov/grants/peer_review_process.htm#scoring.

MEETING ROSTER

The roster for this review meeting is displayed as an aggregated roster that includes reviewers from multiple CSR Special Emphasis Panels of the Risk, Prevention and Health Behavior Integrated Review Group
for the 2021/05 council round.
This roster for CSR is available at:
http://public.era.nih.gov/pubroster/Reports?DOCTYPE=SEP&DESFORMAT=PDF&AGENDA_SEQ_NUM_P=411991
