# Supplementary figures and images for: Development and Evaluation of a Web-Based Platform for Personalized Educational and Professional Assistance for Dementia Caregivers: Proposal for a Mixed Methods Study
Source: JMIR Res Protoc. 2024 Aug 7;13:e64127. doi: 10.2196/64127 (PMC11339571; doi:10.2196/64127)

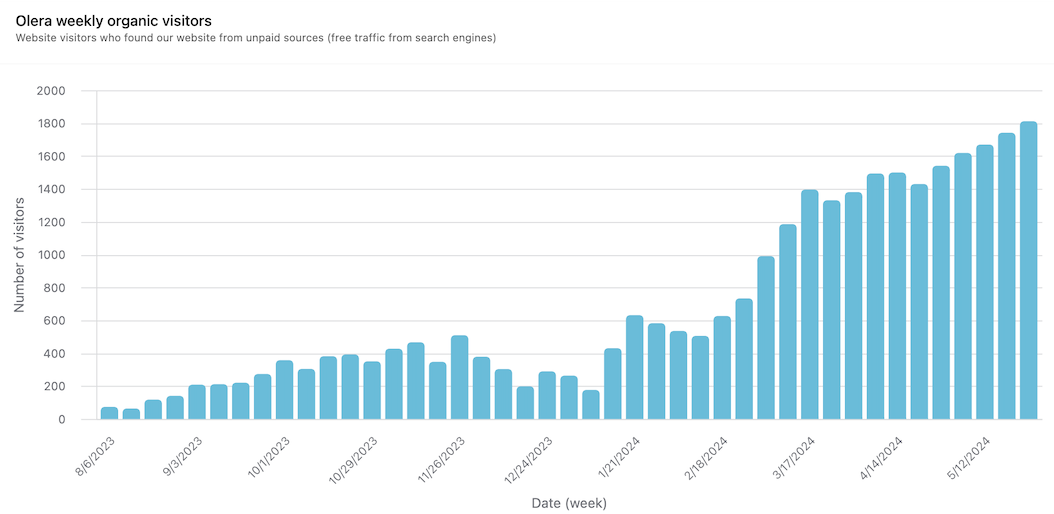

Supplement: Multimedia Appendix 2 [file resprot_v13i1e64127_app2.png]

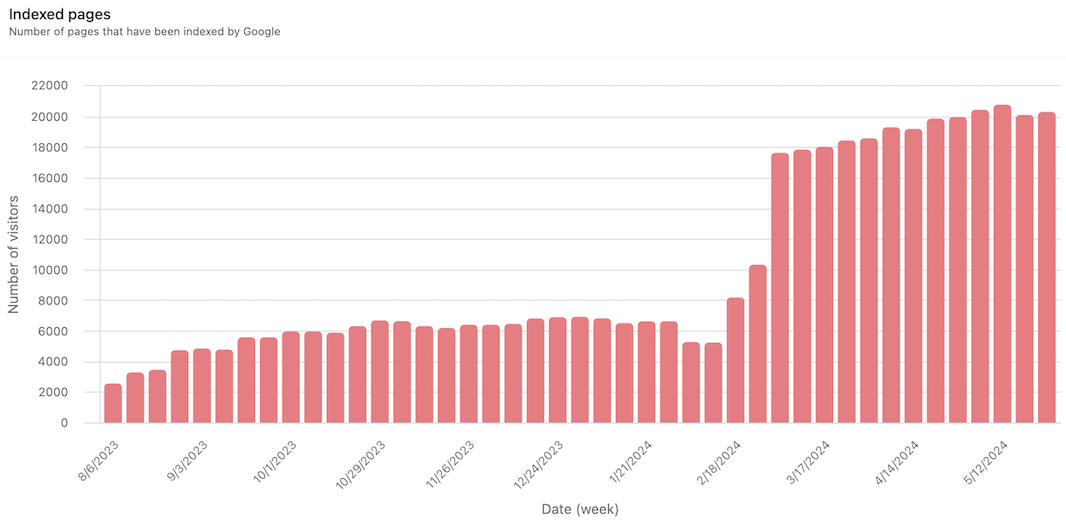

Supplement: Multimedia Appendix 3 [file resprot_v13i1e64127_app3.png]

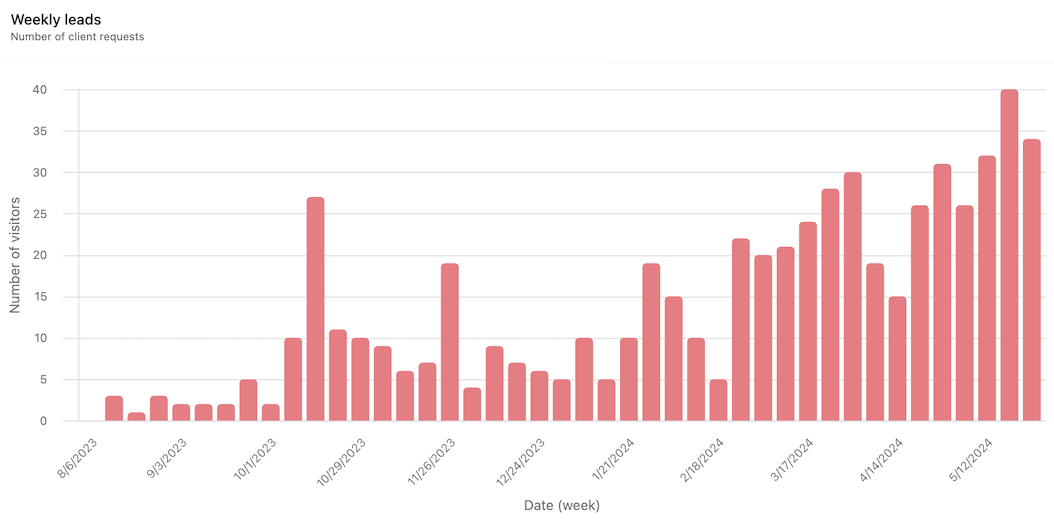

Supplement: Multimedia Appendix 4 [file resprot_v13i1e64127_app4.png]
